# Hf-Nd isotopic variability in mineral dust from Chinese and Mongolian deserts: implications for sources and dispersal

**DOI:** 10.1038/srep05837

**Published:** 2014-07-25

**Authors:** Wancang Zhao, Youbin Sun, William Balsam, Huayu Lu, Lianwen Liu, Jun Chen, Junfeng Ji

**Affiliations:** 1 Key Laboratory of Surficial Geochemistry, Ministry of Education; School of Earth Sciences and Engineering, Nanjing University, Nanjing 210023, China; 2 Ningxia Geophysical and Geochemical Exploration Institute, Yinchuan 710001, China; 3 SKLLQG, Institute of Earth Environment, Chinese Academy of Sciences, Xi'an 710075, China; 4 Department of Earth Sciences, Dartmouth College, Hanover, New Hampshire, USA; 5 School of Geographic and Oceanographic Sciences, Institute for Climate and Global Change Research, Nanjing University, Nanjing 210023, China

## Abstract

Mineral dust provenances are closely related to the orogenic processes which may have distinct Hf-Nd isotopic signatures. Here we report the clay-sized (<2 μm) Hf-Nd isotope data from Asian dust sources to better constrain the source and transport dynamics of dust deposition in the North Pacific. Our results show that there is a more positive radiogenic Hf isotopic composition with clay-sized fractions than the corresponding bulk sample and a decoupling of the Hf-Nd couplets in the clay formation during the weathering process. The clay-sized Hf-Nd isotopic compositions of the desert samples from the Sino-Korean-Tarim Craton (SKTC) are different from those of the Gobi and deserts from the Central Asian Orogeny Belt (CAOB) due to varying tectonic and weathering controls. The Hf-Nd isotopic compositions of dust in the North Pacific central province (NPC) match closely with those from the Taklimakan, Badain Jaran and adjacent Tengger deserts, implying that the NPC dust was mainly transported from these potential sources by the westerly jet. Our study indicates that dusts from the CAOB Gobi deserts either didn't arrive in NPC or were quantitatively insignificant, but they were likely transported to the North Pacific margin province (NPM) by East Asian winter monsoon.

Mineral dust accounts for more than 50% of the atmospheric dust loading, with the clay fraction (<2 μm) comprising about half of mineral dust^1^. It plays an important role in the marine and terrestrial geochemical cycles and impacts global climate by scattering and absorbing solar radiation, changing cloud properties, affecting bio-geochemical cycles and providing important surfaces for atmospheric reactions in the earth–atmosphere–ocean system^2^,^3^,^4^,^5^,^6^. Fine Asian dust (<2.5 μm) is a consistent component of the troposphere over the eastern Pacific and western North America^7^. Asian dust is the second largest source of dust on Earth and has been studied intensively over the past decade, especially with ^87^Sr/^86^Sr and ^143^Nd/^144^Nd ratios of <75 μm silicate particles which are seen as a powerful tool to identify source areas^8^,^9^,^10^,^11^,^12^. However, the isotopic geochemistry of the clay-sized fraction of Asian dust has not been studied; especially the clay-sized Hf isotopic fingerprints of its provenance have not been reported.

Hf-Nd isotopes have been widely used for provenance research in the field of global geochemical cycles^13^,^14^,^15^,^16^. Hf and Nd isotopes plot with a single “mantle-crust” or terrestrial array in igneous and clastic rocks, indicating that Hf and Nd isotopes are coupled during processes that operate the Earth's and crust. Asian eolian dust in the Pacific Ocean is offset towards more radiogenic Hf from the global silicate earth array^17^. The recent conclusion that the oceanic ferromanganese crusts and terrigenous clays, deviate significantly from the Terrestrial Array towards higher ε*_Hf_* values relative to their ε*_Nd_* values, mainly result from the incongruent behavior of Hf during continental weathering^18^,^19^,^20^,^21^,^22^,^23^. This Hf-Nd decouple is still under debate^18^,^21^,^24^,^25^,^26^. Furthermore, eolian dust input with high ε*_Hf_* was only discovered in recent years^17^,^27^,^28^,^29^,^30^.

Biscaye et al.^31^ suggested that the probable source area of the GISP2 dusts was in East Asia by comparing the Nd isotopes of fine fraction (<5 μm) dust particles extracted from Asian Gobi/sandy deserts and Greenland ice cores. Previous studies have confirmed that coarse silt grains are only transported short distances (<3 km) through saltation and short-term suspension due to their greater density. The smaller the dust particles are, the longer they stay in suspension in atmosphere and the further they will be transported. However, clay-sized particles might be lifted to the upper troposphere (>8 km) and transported over a long distance by the westerly jet^32^,^33^,^34^ and clay minerals were relatively enhanced in samples in remote locations^35^. In particular, compared to the non-clay fraction (>2 μm), the clay-sized fraction (<2 μm) has unique minerals phases (dominant by clay minerals like illite, kaolinite, chlorite and smectite) and is removed from the atmosphere by wet deposition (precipitation scavenging)^36^,^37^,^38^. Thus, the clay-sized isotopic fingerprints from Asian deserts may be ideal targets not only for provenance tracing of long-distance transported mineral dust, but also provide an unparalleled window for understanding the global dust cycle, especially, eolian dust preserved in deep-sea sediments.

To better understand how the Asian dust cycle influences marine sediments and sea water in North Pacific Ocean, we conducted a detailed investigation on the clay-sized Hf-Nd isotopic compositions from the Gobi/sandy deserts in North China and neighboring Mongolia. Our objectives are to address the following questions. 1) What controls the Hf-Nd isotopic composition of clay-sized fractions within desert sands? 2) What are the general characteristics of Asian dust and how do those characteristics differ from other dust sources? 3) What are implications for the source and transport pathway of eolian dusts in the North Pacific Ocean?

## Results

**Figure 1:**
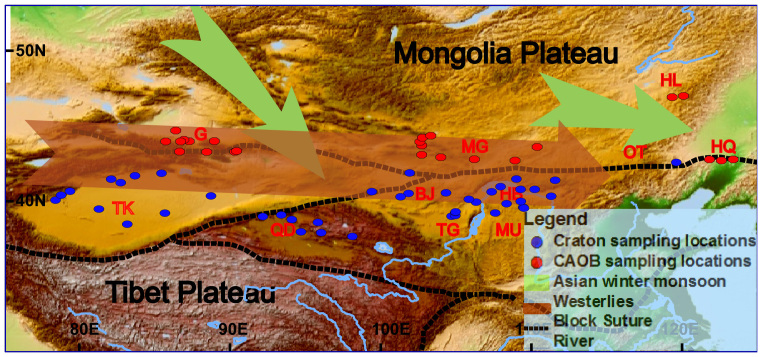
Location Map of this study. Map showing the distribution of Gobi and sandy deserts in northern China and southern Mongolia, including the Gobi desert in southern Mongolia (MG) and northern China, Tengger (TG), Badain Juran (BJ), Mu Us (MU) and Hobq (HB) sandy deserts in northern China, Taklimakan (TK) and Gurbantunggut (G) desert in northwestern China, Qtingdag (OT), Hulun Buir (HL) and Horqin (HQ) sandy lands in northeastern China, and Qaidam Desert (QD) in the northern Tibetan Plateau. Both Red and blue dots indicate location of surface desert samples studied. The figure was generated using ARC-GIS (http://www.esri.com/software/arcgis/) and the map will not have a copyright dispute.

**Figure 2:**
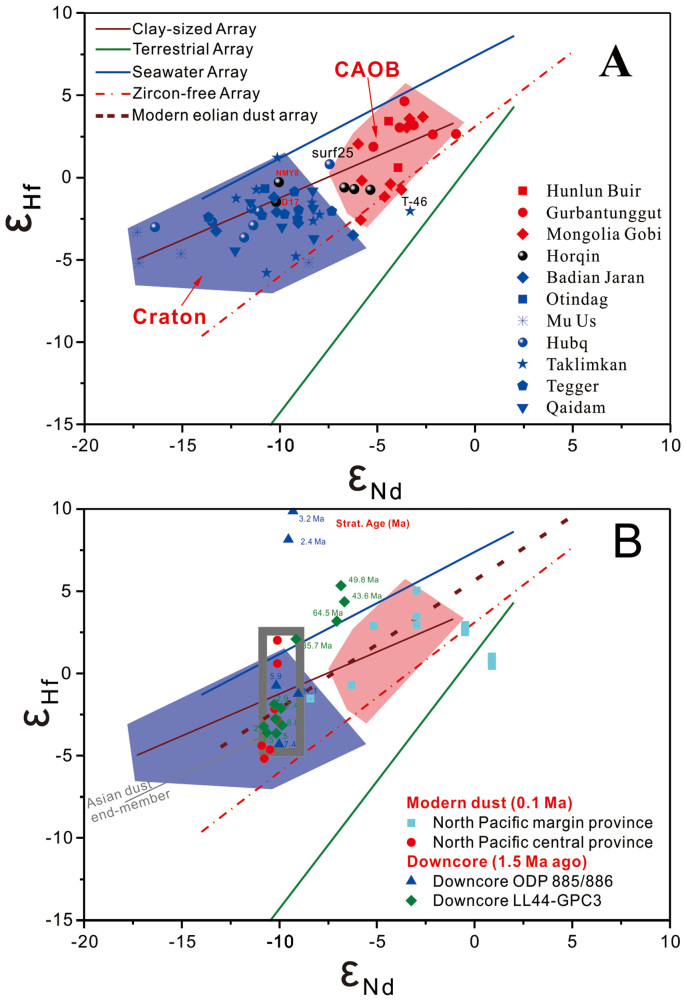
The diagram for the clay-sized ε_Hf_ and ε_Nd_ from all major Chinese deserts and the Mongolia Gobi desert. The clay sized array is shown through the regression with all clay-sized desert Hf-Nd isotopic data: ε_Hf_ = (0.45 ± 0.04) × ε_Nd_ + (2.81 ± 0.35) (R = 0.80). The new Terrestrial Array (ε_Hf_ = 1.55 ε_Nd_ + 1.21), Zircon-free Array (ε_Hf_ = 0.91 ε_Nd_ + 3.1) and Seawater Array (ε_Hf_ = 0.62 ε_Nd_ + 5.27) are from Vervoort (2011)^21^, Bayon(2009)^23^ and David (2001)^48^, respectively. The ε_Hf_-ε_Nd_ diagram compares the desert clays with the Asian eolian dust extracted from the North Pacific Ocean^17^.

**Figure 3:**
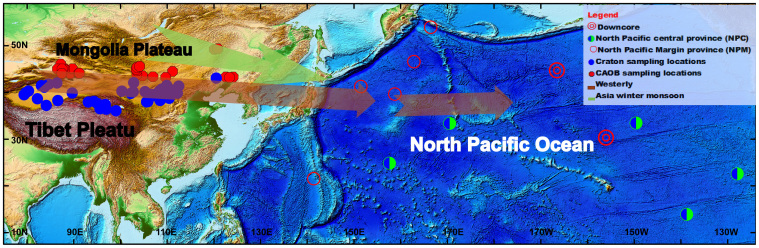
Dust sources and dispersal patterns of Asian dust. Red and blue circles indicate location of eolian dust from bulk pelagic sediments in the North Pacific analyzed by Pettke^17^. North Pacific margin provenance (NPM, red circle) and North Pacific center provenance (NPC, half green circle) indicate the locations of Asian dust in North Pacific Ocean. Downcore ODP 885/886 and LL44-GPC3 are indicated by double red circles and dust transmission pathways are shown by green and sepia arrows. The figure was generated using ARC-GIS (http://www.esri.com/software/arcgis/) and the map will not have a copyright dispute.

Sampling sites of desert sands are situated on the Sino-Korean-Tarim Craton^39^ (SKTC, including North China Craton^9^,^40^) and the Central Asian Orogeny Belt (CAOB)^41^,^42^. The study areas include the Chinese deserts, Mongolian Gobi and northwest Pacific Ocean, are shown in Figure 1 and Figure 3. The Hf and Nd isotopic data of the clay-sized fractions of the Chinese deserts and the Mongolian Gobi are presented in Figure 2A (see Table S1 in Supplementary information). The Chinese deserts have ε_Nd_ values ranging from −17.30 to 0.98 (mean = −8.40) and ε_Hf_ from −5.94 to 4.63 (mean = −0.97). The Mongolian Gobi is more radiogenic in Nd and Hf isotope compositions which ranges from −5.99 to −2.67 (mean = −4.43; n = 9) and from −2.56 to 3.68 (mean = 0.81; n = 9), respectively. It is clear that the clay-sized fractions have higher radiogenic Hf isotopic composition than silt-to-sand silicate fractions (>2 μm) (see Figure S1 and Table S2 in Supplementary information). The ε_Nd_ (ε_Nd_ = −5.4) of <2 μm fraction in sample BT-46 are similar to those of <75 μm fractions (ε_Nd_ = −5.6)^8^, while the ε_Hf_ of >2 μm fractions of BT-46 have ε_Hf_ values ranging from −21.01 to −6.72 (see Figure S2 and Table S3 in Supplementary information). The Sm/Nd values both >2 μm and <2 μm fraction are in good agreement with nearly constant Sm/Nd ratio (Sm/Nd = 0.18) (see Table S3 in Supplementary information). However, Lu/Hf ratio (Lu/Hf_<2 *μm fraction*_ = 0.1) of the <2 μm fraction is much higher than that (Lu/Hf_>2 *μm fraction*_ = 0.05) of the >2 μm fraction (Supplementary information).

The clay-sized Hf-Nd isotopic compositions are relatively consistent for samples taken from individual deserts (Figure 1) and samples derived from the same tectonic terrane display common characteristics (Figure 2A). For example, samples from the Central Asian Orogeny Belt (CAOB, Altay Mts.-Tianshan Mts.-Yin Mts.-Daxinganling Mts) were easily differentiated from the Sino-Korean-Tarim Craton (SKTC) desert samples based on their Hf-Nd isotopic composition, supporting two separate isotopic provinces as reported before^8^,^43^:

1. The CAOB, including the Mongolian Gobi, Gubanunggut Deserts, the Hunlun Buir sandy land, and the west Horqin sandy land, with ε*_Hf_* = −2.58 to 3.68 (mean = 0.86, n = 30) and ε*_Nd_* = −10.19 to −0.98 (mean = −5.91, n = 25).
2. The STKC, including the Taklimakan, Qaidam, Badaim Jaran, Tengger and Mu Us Deserts, and the east Hobq sandy land, with ε*_Hf_* from −5.94 to 1.20 (mean = −2.4, n = 41) and ε*_Nd_* from −17.30 to −6.24 (mean = −10.4, n = 37).

## Discussion

### Tectonic controls on the clay-sized Hf-Nd isotopes

The Hf-Nd isotopic signatures of the Chinese and Mongolia Gobi Deserts are consistent with previous Sr-Nd isotopic observations, suggesting that the isotopic composition of deserts is closely related to the tectonic setting of the surrounding mountains^8^. Hf isotopic systematics can distinguish between orogenic processes dominated by the generation and reworking of continental crust and those dominated by additions of juvenile crust^16^. The geological setting of Paleozoic exposures clearly shows that there are two first-order geological and tectonic units in the research area (i.e., the CAOB and the SKTC)^41^,^42^ (Fig. 2A). The Gobi Desert, Gubanunggut Desert and Hulun Buir Sandy land are on the CAOB, whereas the Taklimakan, Qaidam, Badaim Jaran, Tengger and Mu Us Deserts are on the SKTC. The Tarim, Qaidam and Alxa blocks belonged to the Sino-Korean tectonic domain during the Archean-Mesoproterozoic^39^. The relatively high clay-sized ε*_Hf_* – ε*_Nd_* value within CAOB indicates that the clay-sized fraction is generated from CAOB juvenile continental crust, which was formed by the collision between the Siberian Plate and the southern blocks during the early stages of the orogeny about 1.0 Ga and continued to about 250 Ma^44^. The clay-sized ε*_Hf_* – ε*_Nd_* values controlled by CAOB are higher than SKTC terrane, whereas the variability of both ε*_Hf_* and ε*_Nd_* in the CAOB are smaller than the corresponding SKTC ε*_Hf_* and ε*_Nd_*. There are two obvious end-members easily discerned from present clay-sized ε*_Hf_* – ε*_Nd_* compositions shown in Figure 2A. The clay-sized fractions derived from the old continental shield produce the lowest ε*_Hf_* and ε*_Nd_* values, especially the samples from the Mu Us and Hobq Deserts. The isotopic regions are consistent relative to the clay-sized ε*_Hf_* – ε*_Nd_* values from deserts of the same geologic setting, suggesting that not all the isotopic differences are caused entirely by the heterogeneity of material at their source. Geologically, blocks and/or cratons formed the Chinese continent through multiple collisions and aggregation^41^. The clay-sized Hf-Nd isotopes of the Qaidam Desert were simliar to the SKTC terrane, we thus conclude that the Qaidam basin was attributed to SKTC, even if the Qaidam basin was influenced by the proximity to Altunshan Fault, Tarim craton and Central China Orogen^42^,^45^.

It is noteworthy that samples D17 and Nmy-8 are SKTC end members whereas the other Horqin samples belong to CAOB end members. These are the few exceptions to the geographic distribution of the Hf-Nd isotopic composition, although both D17 and Nmy-8 are from the southern-most edge of Horqin sandy land which is located in the SKTC (Figure 2A). One possible reason for these exceptions is that the boundary between different tectonic domains (Figure 1, Block Suture) may run through the southern part of Horqin sandy land from west to east^41^, and in term of source materials, both D17 and Nmy-8 may actually belong to SKTC. Geographically, both D17 and Nmy-8 belong to CAOB, but their source may be the mountainous area of North China Craton as assessed by comparing clay-sized ε*_Hf_* – ε*_Nd_* values. In fact, the sand sediments in the southern Horqin sandy land appears to be transported directly from the northern mountainous margin of North China Craton by rivers^46^. However, the other two exception samples (T46 and Surfer25), which are located on the northeast of Taklimakan Desert and the northern edge of Hubq Desert between CAOB and SKTC tectonic domain, respectively, do not fall within the SKTC end-member but close to CAOB end member edge, implying that they were controlled by CAOB and SKTC tectonic domains. Instead they reflect the influence of surface transport causing the isotopes to be skewed toward the CAOB by near-surface northwesterly wind^47^.

### Clay array and continental weathering

Regression of all the clay-sized data yields a clay array: ε*_Hf_* = (0.45 ± 0.04) × ε*_Nd_* + 2.81 ± 0.35 (R = 0.80, Figure 2A). The clay array displays a broad band extending between the Seawater array^22^,^48^ and the new terrestrial array^21^. The offset of the clay-sized Hf and Nd isotopic composition from the terrestrial array toward the seawater array can be generated by incongruent weathering of continental rocks, which is known as “zircon effect”^24^,^25^,^49^. The zircons, with low ε*_Hf_*, have relatively high Hf concentrations and indestructibility, and contain large amounts of unradiogenic Hf, causing relatively radiogenic Hf to enter weathering products and/or fine-grained sediments. Thus, clay minerals, the weathering products of continental rocks, are expected to be more radiogenic that primary rocks or bulk sediments. One would explain the elevated radiogenic Hf composition of the clay fraction by the zircon-free effect (mineralogical sorting or grain size effect), because the clay fractions are too fine (<2 μm) to contain any zircon. However, the clay array is above and underscored by the zircon-free sediment array^23^, suggesting that the zircon-free effect alone is insufficient to generate the clay ε*_Hf_* – ε*_Nd_* relationships because the clay-sized fractions contain relatively more radiogenic Hf than fine-grained sediments (zircon-free sediment). During the weathering process, clay minerals incorporate and/or adsorb the incongruent released radiogenic Hf to form the decoupling of the clay-sized Hf and Nd isotopic compositions, which are determined by both the weathering regime and source provenance.

This Hf-Nd isotopic decoupling is attributed to the different Goldschmidt behavior of Hf and Nd during weathering. Hf is both similar to REE and Zr, whereas Nd is one of the REE. REE barely fractionate during weathering and have been used for studying the provenance of detrital sediments. More specifically, ratios such as REE ratio and Nd isotopic compositions of weathered material, are considered to represent the compositions of source rocks^8^,^18^, whereas Hf's behavior was affected by Zr, showing a decoupling with REE. This can be tested by the difference of Lu/Hf and Sm/Nd ratios for size fractions (Lu, Sm and Nd are REE). The <2 μm fraction shows a higher Lu/Hf ratio (0.1) than the >2 μm fraction (0.05), whereas the Sm/Nd ratio remains the same (0.18) (Table S3, and Figure S3 in Supplementary information), suggesting the difference or decoupling of Hf to REE during weathering process.

The decoupling of clay-sized Hf-Nd isotopic compositions may explain the different Hf-Nd correlation patterns between SKTC and CAOB. It is noted that the SKTC clay-sized array has a higher radiogenic Hf isotopic composition for its corresponding Nd isotopic composition than the CAOB array. The Hf isotope signatures from SKTC also show more scatter. This is attributable to the SKTC being older and thereby containing less radiogenic Nd than CAOB as demonstrated above. The clay reservoir from the SKTC had more time to produce more radiogenic Hf isotopes than that from the CAOB. This further suggests that the Hf isotope fractionation between clay and crustal arrays is larger than the older the source rocks are.

### Implication of Asian dust transported to Northern Pacific Ocean

The Chinese deserts and Mongolia Gobi are major Asian dust sources on a hemispheric scale^9^,^31^,^50^. Studies of dust tracers and satellite imagery of the tracks of dust transport unambiguously show that eolian dust from the Asian desert regions are transported globally; some are deposited in the central north Pacific Ocean^50^,^51^,^52^,^53^. Grain size of eolian dust extracted from LL44-GPC3 is indeed less than 2.4 μm^51^.

Pettke et al. (2002)^17^ chemically isolated the Asian dust components from bulk pelagic sediments in the north Pacific Ocean, and reported the Hf-Nd isotopes of modern dust (about 0.1 Ma): −3.8 > ε*_Nd_* > −10.9 and −16.2 > ε*_Hf_* > −4.5, which encompassed the range observed for the Neogene time series of Ocean Drilling Program (DSDP) 885/886 and LL44-GPC3 (Figure 2B). It was suggested that Hf-Nd isotopic results are consistent with a dominantly binary mixture of dust contributed from island arc volcanic material and dust from central Asian deserts^24^. Comparison of our clay-sized desert Hf-Nd isotopic data with the dust records in North Pacific Ocean produces significant implications.

The majority of Hf-Nd isotopic data^17^ plot within the SKTM and CAOB areas in the Hf-Nd isotope space, while a few >25 Ma samples from downcore LL44-GPC3, A-2H-5 (2.4 Ma) and A-3H-1 (3.2 Ma) samples from 885/886 plot above the seawater array (Figure 2B). The Hf-Nd isotopic correlation line for modern dust (ε*_Hf_* = 0.78ε*_Nd_* + 5.66) is very close to our clay-sized array (Figure 2B). This consistency may indicate that the clay-sized dust deposited in the North Pacific Ocean was predominantly derived from the Northwest China and Mongolia deserts. This could explain the flat Hf-Nd isotopic correlation and the variable and radiogenic ε_Hf_ values of the North Pacific modern dusts^17^ that is characteristic of the clay-sized fractions of the Asian deserts.

The Hf-Nd isotopic values of the Asian dust end-member were reported^24^ as −9.0 > ε*_Nd_* > −10.8 and 2.5 > ε*_Hf_* > −4. These isotopic values are from the SKTC Hf-Nd isotope space (Figure 2B) and match closely with Hf-Nd isotope data from the Taklimakan, Badain Jaran and adjacent Tengger deserts. Because this value is determined by dusts chemically isolated from the North Pacific central province (NPC) sediments, this suggests that modern dusts deposited in the NPC were mainly from these deserts and that dusts from the Mongolian deserts were volumetrically inconsequential. Satellite observations of certain dust storm trajectories might support the above scenarios. For example, dust originating from the Taklimakan desert was observed lofted to the upper troposphere, around 8–10 km, and is deposited largely over the North Pacific^7^. In contrast, remotely sensed dust observations suggest that dust from the Mongolian Gobi desert was carried in a northeastward trajectory as it leaves the Asian continent, then travels eastward and is deflected to the south near the Aleutians before it enters the western American coast^54^. These different dust transport pathways may indicate that the clay-sized Hf-Nd isotopic signal entrained by different prevailing winds, such as winter monsoon and westerly.

The Hf-Nd isotopic data of eolian dusts isolated from pelagic sediments in NPM plot in or near the CAOB area in the Hf-Nd isotopic correlation diagram (Figure 2B). This may imply that modern eolian dusts deposited in the NPM may have a dominant CAOB origin besides the commonly accepted origin of the binary mixture of dust contributed from island arc volcanic material and Asian dust with an SKTC origin as discussed above. Based on the comparison with the ε*_Hf_* – ε*_Nd_* from the time series of Ocean Drilling Program (DSDP) 885/886 and LL44-GPC3, the source of Neogene dust in NPC may come from SKTC. Our clay-sized Hf-Nd isotopic signals from major Asian Gobi/sandy deserts indicate that sources and dispersal patterns of dust deposits in the NPC and NPM are spatially different (Figure 3).

## Methods

Samples of surface sand were collected from all the potential sources of Asian dust by first removing the top 5 cm and then sampling to a depth of 10 ~ 20 cm. The sampled deserts and sandy lands include the Hulun Buir and Horqin sandy lands in northeastern China, Gurbantunggut and Taklimakan Deserts in northwestern China, the Qaidam Basin in the northern Tibetan Plateau, the Badain Jaran and Tengger Desert on the Alxa Plateau, the Hobq Desert and Mu Us Desert on the Ordos Plateau, and the Gobi Desert on the Mongolian plateau. The exception to our sampling routine was the Mongolian Gobi desert samples which were collected by scratching off 1 ~ 2 cm thick clay mud crust^55^. According to the geological setting of Paleozoic exposures, the Chinese and Mongolian deserts are on the Sino-Korean-Tarim Craton^39^ (SKTC, including North China Craton^9^,^40^) and the Central Asian Orogeny Belt (CAOB)^41^,^42^.

In order to isolate just the clay sized silicate mineral fraction for Hf analysis, and organic matter and carbonate were removed: organic matter was removed with excess hydrogen peroxide (30%) overnight and then a decarbonation step was carried out using excess 1M acetic acid for 10 hours in order to eliminate the influence of secondary carbonate on Hf isotopic composition. The samples were subsequently rinsed at least three times with MilliQ water to completely remove major ions and soluble salts. Different fractions were extracted by sieving the ultrasonically dispersed samples in mesh with MilliQ water, and the <2 μm particles were separated based on the Stokes' Law and then were recovered by centrifuging^56^. The samples were subsequently rinsed at least three times with MilliQ water to completely remove major ions and soluble salts.

The Hf-Nd isotopic ratios of the extracted clay-sized fractions were measured with a Thermo Fisher Scientific Neptune MC-ICP-MS at the State Key Laboratory for Mineral Deposits Research, Nanjing University. These samples were prepared as follows: First, sample digestion. 100 mg of the dry silicate residue was totally dissolved with HF–HClO_4_ mixture in a steel jacketed autoclaves at 180 ~ 200°C for 72 hours^57^, while 100 mg of clay-sized fractions were digested with a mixture of HF–HClO_4_ at 110 ~ 140°C for 72 hours. Second, the purification for Hf and Nd with ion chromatography. The Hf analysis used a modified version of the method of Yang et al (2010)^57^. Modifications include dissolving the samples in an HF–HClO_4_ mixture and separating them by chromatographic extraction through an cation exchange resin(Bio-Rad 50 WX8 resin^+^ Eichrom® Ln-Spec resin. Hafnium was separated from matrix by ion exchange procedures using Eichrom® Ln-Spec resin, These detailed analytical procedure for the Hf isotopic measurement can be seen elsewhere^57^. Nd was then separated and purificated by ion exchange procedures followed the detailed method from Pu et al^58^. All chemical digestion and purification were carried out in Class 100 ultra-clean laboratory. The total procedure blank for Lu, Hf, Sm and Nd were less than 10 pg, 50 pg, 50 pg and 60 pg, respectively, and thus negligible.

The mass spectrometric analyses were performed in Class 1000 clean laboratories. The JMC-475 Hf standard^59^ (^176^Hf/^177^Hf = 0.282160 ± 0.00005, 200 ppb) was analyzed to provide a calibration value for the standard: ^176^Hf/^177^Hf = 0.282161 ± 0.000004 (n = 20, 2σ). The JNDi-1 Nd standard gave an average value of ^143^Nd/^144^Nd = 0.512118 ± 0.000005 (50 ppb), which is similar to the referenced value of ^143^Nd/^144^Nd = 0.512115 ± 000007 (n = 15, 2σ)^60^. Instrumental mass bias was corrected for using ^146^Nd/^144^Nd ratio of 0.7219 and ^179^Hf/^177^Hf ratio of 0.7325. The external reproducibility of the ^176^Hf/^177^Hf and ^143^Nd/^144^Nd ratios was estimated from repeated measurements of JMC 475 and JNDi-1 Nd standard solutions every tenth sample. The relative standard deviations are better than 9 × 10^−6^. Epsilon Hf and Nd values were calculated using chondritic values of ^176^Hf/^177^Hf = 0.282785 and ^143^Nd/^144^Nd = 0.512630^61^. Replicates for both ε*_Hf_* and ε*_Nd_* were processed and yielded an external reproducibility of better than ± 0.1 (2σ) for ε*_Nd_* and ± 0.1 (2σ) for ε*_Hf_*.

## Supplementary Material

Supplementary Information Supplementary information

## References

[b1] Murray B. J., O'Sullivan D., Atkinson J. D. & Webb M. E. Ice nucleation by particles immersed in supercooled cloud droplets. Chem Soc Rev 41, 6519–6554 (2012). 22932664 10.1039/c2cs35200a

[b2] Maher B. A. *et al.* Global connections between aeolian dust, climate and ocean biogeochemistry at the present day and at the last glacial maximum. Earth-Sci Rev 99, 61–97 (2010).

[b3] Ferrat M. *et al.* Improved provenance tracing of Asian dust sources using rare earth elements and selected trace elements for palaeomonsoon studies on the eastern Tibetan Plateau. Geochim Cosmochim Ac 75, (2011).

[b4] Lambert F. *et al.* The role of mineral-dust aerosols in polar temperature amplification. Nat Clim Change 3, 487–491 (2013).

[b5] Creamean J. M. *et al.* Dust and biological aerosols from the Sahara and Asia influence precipitation in the western U.S. Science 339, 1572–1578 (2013). 23449996 10.1126/science.1227279

[b6] Cziczo D. J. *et al.* Clarifying the dominant sources and mechanisms of cirrus cloud formation. Science 340, 1320–1324 (2013). 23661645 10.1126/science.1234145

[b7] VanCuren R. A. & Cahill T. A. Asian aerosols in North America: Frequency and concentration of fine dust. J Geophys Res-Atmos 107, (2002).

[b8] Chen J. *et al.* Nd and Sr isotopic characteristics of Chinese deserts: Implications for the provenances of Asian dust. Geochim Cosmochim Ac 71, 3904–3914 (2007).

[b9] Chen J. & Li G. J. Geochemical studies on the source region of Asian dust. Sci China Earth Sci 54, 1279–1301 (2011).

[b10] Grousset F. E. & Biscaye P. E. Tracing dust sources and transport patterns using Sr, Nd and Pb isotopes. Chem Geol 222, 149–167 (2005).

[b11] Li G. J., Chen J., Ji J. F., Yang J. D. & Conway T. M. Natural and anthropogenic sources of East Asian dust. Geology 37, 727–730 (2009).

[b12] Meyer I., Davies G. R. & Stuut J.-B. W. Grain size control on Sr-Nd isotope provenance studies and impact on paleoclimate reconstructions: An example from deep-sea sediments offshore NW Africa. Geochem. Geophys. Geosyst. 12, Q03005 (2011).

[b13] Frank M. Radiogenic isotopes: Tracers of past ocean circulation and erosional input. Rev. Geophys. 40, 1001 (2002).

[b14] Bau M. & Koschinsky A. Hafnium and neodymium isotopes in seawater and in ferromanganese crusts: The “element perspective”. Earth Planet Sc Lett 241, 952–961 (2006).

[b15] Dhuime B., Hawkesworth C. J., Storey C. D. & Cawood P. A. From sediments to their source rocks: Hf and Nd isotopes in recent river sediments. Geology 39, 407–410 (2011).

[b16] Collins W. J., Belousova E. A., Kemp A. I. S. & Murphy J. B. Two contrasting Phanerozoic orogenic systems revealed by hafnium isotope data. Nature Geosci 4, 333–337 (2011).

[b17] Pettke T., Lee D.-C., Halliday A. N. & Rea D. K. Radiogenic Hf isotopic compositions of continental eolian dust from Asia, its variability and its implications for seawater Hf. Earth Planet Sc Lett 202, 453–464 (2002).

[b18] Bayon G. *et al.* The control of weathering processes on riverine and seawater hafnium isotope ratios. Geology 34, 433–436 (2006).

[b19] Rickli J. *et al.* Controls on the incongruent release of hafnium during weathering of metamorphic and sedimentary catchments. Geochim Cosmochim Ac 101, 263–284 (2013).

[b20] Garçon M., Chauvel C., France-Lanord C., Huyghe P. & Lavé J. Continental sedimentary processes decouple Nd and Hf isotopes. Geochim Cosmochim Ac 121, 177–195 (2013).

[b21] Vervoort J. D., Plank T. & Prytulak J. The Hf–Nd isotopic composition of marine sediments. Geochim Cosmochim Ac 75, 5903–5926 (2011).

[b22] Albarede F., Simonetti A., Vervoort J. D., Blichert-Toft J. & Abouchami W. A Hf-Nd isotopic correlation in ferromanganese nodules. Geophys Res Lett 25, 3895–3898 (1998).

[b23] Bayon G. *et al.* Hf and Nd isotopes in marine sediments: Constraints on global silicate weathering. Earth Planet Sc Lett 277, 318–326 (2009).

[b24] Patchett P. J., White W. M., Feldmann H., Kielinczuk S. & Hofmann A. W. Hafnium/rare earth element fractionation in the sedimentary system and crustal recycling into the Earth's mantle. Earth Planet Sc Lett 69, 365–378 (1984).

[b25] Carpentier M., Chauvel C., Maury R. C. & Mattielli N. The “zircon effect” as recorded by the chemical and Hf isotopic compositions of Lesser Antilles forearc sediments. Earth Planet Sc Lett 287, 86–99 (2009).

[b26] Garçon M., Chauvel C., France-Lanord C., Limonta M. & Garzanti E. Which minerals control the Nd–Hf–Sr–Pb isotopic compositions of river sediments? Chem Geol 364, 42–55 (2014).

[b27] Rickli J. D. The hafnium and neodymium isotopic composition of seawater and rivers PHD thesis, Swiss Federal Institute of Technology in Zurich, (2009).

[b28] Rickli J. *et al.* Hafnium and neodymium isotopes in surface waters of the eastern Atlantic Ocean: Implications for sources and inputs of trace metals to the ocean. Geochim Cosmochim Ac 74, 540–557 (2010).

[b29] Aarons S. M., Aciego S. M. & Gleason J. D. Variable HfSrNd radiogenic isotopic compositions in a Saharan dust storm over the Atlantic: Implications for dust flux to oceans, ice sheets and the terrestrial biosphere. Chem Geol 349–350, 18–26 (2013).

[b30] Lupker M., Aciego S. M., Bourdon B., Schwander J. & Stocker T. F. Isotopic tracing (Sr, Nd, U and Hf) of continental and marine aerosols in an 18th century section of the Dye-3 ice core (Greenland). Earth Planet Sc Lett 295, 277–286 (2010).

[b31] Biscaye P. E. *et al.* Asian provenance of glacial dust (stage 2) in the Greenland Ice Sheet Project 2 Ice Core, Summit, Greenland. J. Geophys. Res. 102, 26765–26781 (1997).

[b32] Stuut J. B., Smalley I. & O'Hara-Dhand K. Aeolian dust in Europe: African sources and European deposits. Quatern Int 198, 234–245 (2009).

[b33] Uno I. *et al.* Asian dust transported one full circuit around the globe. Nature Geosci 2, 557–560 (2009).

[b34] Crouvi O., Amit R., Enzel Y. & Gillespie A. R. Active sand seas and the formation of desert loess. Quaternary Sci Rev 29, 2087–2098 (2010).

[b35] Leinen M., Prospero J. M., Arnold E. & Blank M. Mineralogy of aeolian dust reaching the North Pacific Ocean: 1. Sampling and analysis. Journal of Geophysical Research: Atmospheres 99, 21017–21023 (1994).

[b36] Dong Z. *et al.* Physicochemical characteristics and sources of atmospheric dust deposition in snow packs on the glaciers of western Qilian Mountains, China. Tellus B 66 (2014).

[b37] Twohy C. H. *et al.* Saharan dust particles nucleate droplets in eastern Atlantic clouds. Geophys Res Lett 36, L01807 (2009).

[b38] Osada K. *et al.* Wet and dry deposition of mineral dust particles in Japan: factors related to temporal variation and spatial distribution. Atmos. Chem. Phys. 14, 1107–1121 (2014).

[b39] Wan T. [Tectonics of Archean and Paleoproterozoic (Before 1.8Ga)] in The Tectonics of China [27–49] (Higher Education Press, Beijing, 2012).

[b40] Zhu R.-X., Yang J.-H. & Wu F.-Y. Timing of destruction of the North China Craton. Lithos 149, 51–60 (2012).

[b41] Ren J., Deng P. & Xiao L. Keeping pace with the times to develop the tectonics of China. Earth Science Frontiers 11, 1–7 (2004).

[b42] Pirajno F. [China's Tectonic Framework in the Global Context] in The Geology and Tectonic Settings of China's Mineral Deposits [19–33]. (Springer Netherlands, 2013).

[b43] Li G., Pettke T. & Chen J. Increasing Nd isotopic ratio of Asian dust indicates progressive uplift of the north Tibetan Plateau since the middle Miocene. Geology 39, 199–202 (2011).

[b44] Khain E. The Palaeo-Asian ocean in the Neoproterozoic and early Palaeozoic: new geochronologic data and palaeotectonic reconstructions. Precambrian Res 122, 329–358 (2003).

[b45] Pirajno F. [North China and Tarim Cratonic Blocks] in The Geology and Tectonic Settings of China's Mineral Deposits [35–123] (Springer Netherlands, 2013).

[b46] Xie J. & Ding Z. Compositions of heavy minerals in Northeastern China sandlands and provenance analysis. Science in China Series D: Earth Sciences 50, 1715–1723 (2007).

[b47] Yang X., Zhu B. & White P. D. Provenance of aeolian sediment in the Taklamakan Desert of western China, inferred from REE and major-elemental data. Quatern Int 175, 71–85 (2007).

[b48] David K., Frank M., O'Nions R. K., Belshaw N. S. & Arden J. W. The Hf isotope composition of global seawater and the evolution of Hf isotopes in the deep Pacific Ocean from Fe–Mn crusts. Chem Geol 178, 23–42 (2001).

[b49] White W. M., Patchett J. & BenOthman D. Hf isotope ratios of marine sediments and Mn nodules: evidence for a mantle source of Hf in seawater. Earth Planet Sc Lett 79, 46–54 (1986).

[b50] Fischer E. V., Hsu N. C., Jaffe D. A., Jeong M. J. & Gong S. L. A decade of dust: Asian dust and springtime aerosol load in the U.S. Pacific Northwest. Geophys Res Lett 36, L03821 (2009).

[b51] Rea D. K. The paleoclimatic record provided by eolian deposition in the deep sea: The geologic history of wind. Rev Geophys 32, 159–195 (1994).

[b52] Serno S. *et al.* Eolian dust input to the Subarctic North Pacific. Earth Planet Sc Lett 387, 252–263 (2014).

[b53] Uematsu M. *et al.* Transport of mineral aerosol from Asia Over the North Pacific Ocean. Journal of Geophysical Research: Oceans 88, 5343–5352 (1983).

[b54] Chen K. The northern path of Asian dust transport from the Gobi desert to North America. Atmos. Oceanic Sci. Lett 3, 155–159 (2010).

[b55] Sun Y. *et al.* ESR signal intensity and crystallinity of quartz from Gobi and sandy deserts in East Asia and implication for tracing Asian dust provenance. Geochemistry, Geophysics, Geosystems 14, 2615–2627 (2013).

[b56] Gee G. W. & Or D. 2002. [Particle-size analysis] in Methods of Soil Analysis, Part 4: Physical Methods (eds Dane, J. H. & Topp, C.) [255–293] (Soil Society of America, 2002).

[b57] Yang Y.-h., Zhang H.-f., Chu Z.-y., Xie L.-w. & Wu F.-y. Combined chemical separation of Lu, Hf, Rb, Sr, Sm and Nd from a single rock digest and precise and accurate isotope determinations of Lu–Hf, Rb–Sr and Sm–Nd isotope systems using Multi-Collector ICP-MS and TIMS. Int J Mass Spectrom 290, 120–126 (2010).

[b58] Pu W., Gao J.-F., Zhao K.-D., Ling H.-F. & Jiang S.-Y. Separation Method of Rb-Sr, Sm-Nd Using DCTA and HIBA Journal of Nanjing University (Natural Sciences). 4, 016 (2005).

[b59] Nowell G. M. *et al.* High precision Hf isotope measurements of MORB and OIB by thermal ionisation mass spectrometry: insights into the depleted mantle. Chem Geol 149, 211–233 (1998).

[b60] Tanaka T. *et al.* JNdi-1: a neodymium isotopic reference in consistency with LaJolla neodymium. Chem Geol 168, 279–281 (2000).

[b61] Bouvier A., Vervoort J. D. & Patchett P. J. The Lu–Hf and Sm–Nd isotopic composition of CHUR: Constraints from unequilibrated chondrites and implications for the bulk composition of terrestrial planets. Earth Planet Sc Lett 273, 48–57 (2008).

