# Supplementary material for: Hf-Nd isotopic variability in mineral dust from Chinese and Mongolian deserts: implications for sources and dispersal
Source: Sci Rep. 2014 Jul 25;4:5837. doi: 10.1038/srep05837 (PMC5376165; doi:10.1038/srep05837)

Supplementary information

Table S1

Table S1. Hf-Nd isotope data of clay-sized fractions of the deserts

No.	Tpye	Size	Long (⁰E)	Lat (⁰N)	$^{176}Hf/^{177}Hf$	2δ	ε_{Hf}	$^{143}Nd/^{144}Nd$	2δ	ε_{Nd}
**Hulun Buir Sand**										
**S06**	sand	<2 μm	118.937	49.121	0.282802	4	0.60	0.512428	12	−3.94
**S09**	sand	<2 μm	118.980	49.170	0.282799	2	0.50			
**BT-77**	sand	<2 μm	119.730	49.290	0.282882	2	3.43	0.512403	2	−4.43
**Badain Jaran Desert**										
**BJ-06**	sand	<2 μm	101.580	42.020	0.282726	3	−2.09	0.512109	14	−10.1
**BJ-08**	sand	<2 μm	101.500	40.083	0.282707	5	−2.76	0.512166	8	−9.05
**BJ-10**	sand	<2 μm	99.070	40.250	0.282751	4	−1.20	0.512102	12	−10.3
**Surf47**	sand	<2 μm	103.980	40.150	0.282693	5	−3.25	0.51195	40	−13.2
**Surf44**	sand	<2 μm	100.970	39.770	0.282686	3	−3.50	0.51231	6	−6.24
**Horqin Sandy Land**										
**BT-46**	sand	<2 μm	121.451	43.296	0.282764	4	−0.74	0.512355	8	−5.36
**D14**	sand	<2 μm	123.025	43.292	0.282768	3	−0.60	0.512287	19	−6.69
**D16**	sand	<2 μm	122.240	43.190	0.282765	2	−0.71	0.512313	37	−6.18
**D17**	sand	<2 μm	122.539	42.522	0.282743	4	−1.49	0.512108	16	−10.18
**NMY8**	sand	<2 μm	120.566	42.841	0.282777	6	−0.28	0.512115	63	−10.05
**Gurbantunggut Desert**										
**G-1**	sand	<2 μm	88.130	44.010	0.282838	3	1.87	0.512363	6	−5.21
**G-14**	sand	<2 μm	88.630	45.020	0.282871	4	3.04	0.512432	5	−3.86
**G-20**	sand	<2 μm	86.960	45.010	0.282916	3	4.63	0.512445	42	−3.61
**G-21**	sand	<2 μm	86.600	45.150	0.282885	2	3.54	0.512158	6	−9.21
**G-23**	sand	<2 μm	86.076	46.021	0.282883	5	3.47			
**G-26**	sand	<2 μm	85.400	45.000	0.28286	3	2.65	0.51258	9	−0.98
**G-28**	sand	<2 μm	86.395	45.000	0.282878	6	3.29	0.512462	33	−3.28
**G-30**	sand	<2 μm	86.330	44.040	0.282875	3	3.18	0.512469	10	−3.14
**G-4**	sand	<2 μm	89.990	44.020	0.282859	3	2.62	0.512519	8	−2.17
**G-6**	sand	<2 μm	90.100	44.090	0.282895	3	3.89	0.512109	13	−10.1
**Hunshandake Sandy Land**										
**D05**	sand	<2 μm	119.250	43.015	0.282766	4	−0.67	0.512079	18	−10.75
**Mu Us sandy Land**										
**YCH1**	sand	<2 μm	107.250	38.250	0.282639	2	−5.16	0.512193	5	−8.52
**DBS14**	sand	<2 μm	108.000	39.120	0.282654	3	−4.63	0.511858	11	−15.0
**DBS23**	sand	<2 μm	109.113	38.776	0.28268	6	−3.71			
**DBS29**	sand	<2 μm	108.950	39.340	0.282638	2	−5.20	0.511748	9	−17.2
**DBS39**	sand	<2 μm	109.160	38.700	0.282691	2	−3.32	0.511743	15	−17.3
**Around Hobq Desert**										
**Surf34**	sand	<2 μm	108.650	41.450	0.282703	3	−2.90	0.512048	4	−11.3

**Surf20**	sand	<2 μm	111.220	41.330	0.282709	3	−2.69	0.51194	26	−13.4
**Surf25**	sand	<2 μm	109.870	40.480	0.282808	3	0.81	0.512351	9	−5.44
**Surf39**	sand	<2 μm	107.020	40.230	0.282682	4	−3.64	0.512024	10	−11.8
**Surf19**	sand	<2 μm	110.950	39.830	0.2827	4	−3.01	0.51179	15	−16.39
**DBS01**	sand	<2 μm	107.600	40.720	0.282727	5	−2.05	0.512062	15	−11.0
**DBS10**	sand	<2 μm	108.940	40.470	0.282732	2	−1.87	0.512043	20	−11.4
					**Taklimakan Desert**					
**T-10**	sand	<2 μm	85.350	38.230	0.282765	2	−0.71	0.512072	8	−10.8
**T-14**	sand	<2 μm	82.850	37.180	0.282743	2	−1.49			
**T-25**	sand	<2 μm	82.430	41.110	0.282722	2	−2.23			
**T-30**	sand	<2 μm	80.970	38.610	0.282749	2	−1.27	0.512003	24	−12.2
**T-40**	sand	<2 μm	78.090	39.460	0.282617	2	−5.94			
**T-41**	sand	<2 μm	78.470	39.920	0.282742	3	−1.52	0.512202	10	−8.35
**T-42**	sand	<2 μm	79.050	40.300	0.282649	3	−4.81	0.51216	21	−9.17
**T-45**	sand	<2 μm	81.790	41.440	0.282721	2	−2.26	0.512222	14	−7.96
**T-46**	sand	<2 μm	83.370	41.760	0.282727	2	−2.05	0.51246	30	−3.32
**T-47**	sand	<2 μm	85.120	42.000	0.282621	2	−5.80	0.512083	10	−10.6
**T-5**	sand	<2 μm	88.400	39.860	0.282819	2	1.20	0.512111	4	−10.1
					**Tengger Desert**					
**TG18N**	sand	<2 μm	104.350	37.930	0.282714	3	−2.51	0.512164	46	−9.09
**TG26A**	sand	<2 μm	104.600	38.010	0.282727	3	−2.05	0.512254	17	−7.33
**TG4A**	sand	<2 μm	104.600	38.300	0.282722	4	−2.23	0.51213	18	−9.75
**TG8N**	sand	<2 μm	104.620	38.350	0.282761	2	−0.85	0.512156	9	−9.25
**Surf41**	sand	<2 μm	105.980	39.270	0.283	3	−2.40	0.51193	21	−13.6
**Surf43**	sand	<2 μm	105.500	39.530	0.282729	3	−1.98	0.512167	5	−9.03
					**Qaidam Desert**					
**W-01**	sand	<2 μm	91.840	37.900	0.282739	3	−1.63			
**W-02**	sand	<2 μm	93.070	38.050	0.2827	5	−3.01	0.512123	9	−9.89
**W-03**	sand	<2 μm	93.770	37.630	0.28268	5	−3.71	0.512207	5	−8.25
**W-04**	sand	<2 μm	97.780	36.030	0.282762	3	−0.81	0.512204	20	−8.31
**W-05**	sand	<2 μm	95.710	36.380	0.282732	2	−1.87	0.512205	18	−8.29
**W-06**	sand	<2 μm	94.370	36.460	0.282742	3	−1.52	0.51204	18	−11.51
**W-07**	sand	<2 μm	95.490	37.360	0.282659	2	−4.46	0.512	17	−12.29
					**Mongolian Gobi**					
**mg05**	sand	<2 μm	110.030	44.480	0.282774	5	−0.39	0.512408	6	−4.33
**mg10**	sand	<2 μm	108.569	43.185	0.282843	8	2.05	0.512323	6	−5.99
**mg14**	sand	<2 μm	105.866	43.310	0.282889	3	3.68	0.512493	10	−2.67
**mg18**	sand	<2 μm	103.642	43.506	0.282871	4	3.04	0.512451	1	−3.49
**mg22**	sand	<2 μm	102.377	43.735	0.282886	5	3.57	0.512458	12	−3.36
**mg25**	sand	<2 μm	102.375	44.621	0.282764	9	−0.74	0.512437	20	−3.76
**mg26**	sand	<2 μm	102.260	44.959	0.282712	9	−2.58	0.51233	4	−5.85
**mg27**	sand	<2 μm	102.414	45.349	0.28278	5	−0.18	0.512333	6	−5.79
**mg28**	sand	<2 μm	102.980	45.540	0.282752	4	−1.17	0.512393	11	−4.62

a Errors on $^{176}Hf/^{177}Hf$ and $^{143}Nd/^{144}Nd$ represent within-run uncertainty calculated as 2σ,

SE and expressed as variation in the 6th decimal place.

b ε_{Hf} and ε_{Nd} values are calculated using the chondritic values of $^{176}Hf/^{177}Hf$ = 0.282785 and $^{143}Nd/^{144}Nd$ = 0.512630 (Bouvier et al., 2008).

Table S2

Table S2. The ε_{Hf} values of d**ifferent particle size**

**Sample**	**Type**	**particle size (μm)**	**$^{176}Hf/^{177}Hf$**	**2**	**ε_{Hf}**
**BT-46-2**	sand	250-830	0.282577	8	-7.36
**BT-46-3**	sand	100-250	0.282191	7	-21.01
**BT-46-4**	sand	75-100	0.282537	3	-8.77
**BT-46-5**	sand	43-75	0.282513	3	-9.62
**BT-46-6**	sand	28-43	0.282424	3	-12.77
**BT-46-7**	sand	2-28	0.282595	3	-6.72

Table S3

Table S3 The concentrations(μg/g) of Trace elements and REEs in clay-sized fractions from sands of Chinese deserts and Mongolian Gobi desert

Sample	Sc	Ti	V	Ga	Rb	Y	Zr	Nb	Cs	La	Ce	Pr	Nd	Sm	Eu	Gd	Tb	Dy	Ho	Er	Tm	Yb	Lu	Hf	Ta	Th
BT77	20.7	4469.9	167.7	32.3	153.3	34.0	188.6	15.5	17.0	56.7	105.6	13.3	49.2	9.9	1.9	8.5	1.4	7.6	1.5	4.2	0.7	4.3	0.7	5.7	1.3	21.7
DBS14	28.4	3394.5	166.6	30.9	160.1	26.3	132.5	12.3	15.3	32.6	67.5	7.4	25.8	5.1	1.2	4.5	0.8	4.7	1.0	2.8	0.5	3.2	0.5	4.3	1.1	21.0
DBS29	32.2	3291.7	173.1	32.0	114.9	29.0	106.0	10.6	9.5	71.0	137.1	15.6	55.8	10.4	2.2	8.6	1.4	6.8	1.3	3.5	0.5	3.3	0.5	3.6	0.9	21.9
DBS39	28.7	3050.3	155.5	27.7	105.4	29.7	103.6	11.1	10.9	74.9	154.1	16.8	60.0	11.3	2.2	9.1	1.4	7.2	1.4	3.7	0.6	3.5	0.6	3.5	0.9	22.2
G1	27.8	4195.8	217.1	35.2	148.9	26.3	176.5	12.6	13.6	35.6	80.2	8.3	29.8	6.0	1.3	5.2	0.9	5.4	1.1	3.3	0.6	3.8	0.6	5.6	1.1	20.4
G14	21.7	4568.8	145.7	24.4	129.1	33.5	147.9	15.1	11.1	42.2	93.0	10.3	36.9	7.7	1.5	6.7	1.2	6.5	1.3	3.7	0.6	3.8	0.6	4.6	1.4	19.0
G20	19.7	3910.8	139.3	21.9	106.1	27.8	112.7	13.0	9.7	34.9	66.8	8.2	28.8	5.8	1.2	5.1	0.9	5.1	1.0	2.9	0.5	3.0	0.5	3.6	1.1	12.7
G21	22.0	4683.3	192.0	28.3	68.1	26.9	170.3	13.0	8.8	24.0	47.6	6.1	22.2	4.7	1.1	4.1	0.7	4.4	0.9	2.8	0.5	3.1	0.5	5.0	1.1	9.9
G26	23.0	4212.9	166.8	27.7	149.6	27.0	176.5	11.7	11.2	25.3	52.8	6.2	23.0	4.9	1.1	4.3	0.8	4.8	1.0	3.0	0.5	3.4	0.6	5.4	1.0	14.8
G30	23.6	3462.5	138.7	24.6	136.2	27.5	162.3	10.3	12.1	34.4	67.0	8.3	31.1	6.7	1.4	5.9	1.1	6.0	1.2	3.5	0.6	3.7	0.6	5.3	0.9	17.1
G6	25.7	3961.4	176.6	26.9	170.4	28.4	125.4	15.6	15.4	41.0	81.3	8.7	30.4	6.0	1.2	5.2	0.9	5.1	1.0	3.0	0.5	3.2	0.5	3.8	1.3	26.7
MG18	22.2	4008.9	146.0	30.3	129.4	26.6	134.7	11.5	10.7	39.7	81.9	8.9	31.9	6.2	1.3	5.4	0.9	4.9	1.0	2.8	0.5	2.9	0.5	4.3	1.0	15.8
MG26	16.2	6628.8	121.2	25.8	154.9	30.7	222.0	21.1	13.7	69.2	142.3	16.0	57.2	10.7	2.0	8.6	1.4	7.0	1.4	3.7	0.6	3.8	0.6	6.0	1.6	19.4
MG28	22.4	3978.7	139.7	28.4	89.4	31.7	128.5	9.4	7.0	48.6	95.5	11.6	43.5	8.8	1.9	7.5	1.3	6.8	1.4	3.7	0.6	3.7	0.6	3.9	0.8	16.6
S06	13.8	4522.4	116.7	28.8	164.8	27.9	176.8	16.8	14.2	62.3	159.8	15.5	54.8	10.6	1.9	8.2	1.3	6.5	1.2	3.2	0.5	3.2	0.5	5.3	1.4	25.5
Surf44	22.1	4452.3	276.5	25.9	108.0	22.8	122.7	17.4	8.2	42.2	125.4	8.5	29.5	6.0	1.1	4.9	0.8	4.6	0.9	2.5	0.4	2.6	0.4	4.2	1.6	42.4
T10	26.1	5223.4	194.4	32.0	108.6	28.2	226.7	13.2	10.0	33.2	78.7	7.8	28.8	6.1	1.2	5.4	1.0	5.8	1.2	3.5	0.6	4.0	0.7	6.9	1.1	15.2
T14	21.1	2388.7	131.8	24.0	198.0	26.4	105.6	9.1	26.5	29.3	58.9	6.8	25.2	5.5	1.2	5.1	0.9	5.1	1.0	2.9	0.5	3.1	0.5	3.4	0.8	20.5
T40	21.7	4378.9	164.6	27.4	200.2	27.4	124.5	19.6	17.9	49.6	97.7	11.1	38.2	7.1	1.3	5.8	0.9	4.8	0.9	2.6	0.4	2.7	0.4	3.9	1.6	21.3
T41	26.5	3392.3	187.8	30.3	184.4	25.4	148.3	12.1	16.0	35.7	68.9	7.7	26.8	5.2	1.1	4.4	0.8	4.5	1.0	2.9	0.5	3.3	0.6	4.7	1.0	22.4
T45	22.9	3213.4	177.8	25.0	170.0	25.8	144.5	14.1	15.7	31.0	60.9	6.9	23.9	4.6	1.0	4.0	0.7	3.9	0.8	2.4	0.4	2.7	0.4	4.6	1.1	24.7

T46	25.7	3732.7	180.0	30.7	183.3	26.7	139.8	13.8	15.1	36.1	75.1	7.9	28.2	5.5	1.2	4.9	0.8	4.8	1.0	2.9	0.5	3.2	0.5	4.4	1.2	21.8
T47	24.3	3240.2	173.9	30.6	174.6	26.1	189.6	13.1	17.4	46.9	104.1	10.5	37.2	7.1	1.5	6.0	1.0	5.6	1.1	3.4	0.6	3.9	0.7	6.4	1.2	30.6
TGL11	23.0	4751.7	171.4	27.8	195.5	24.0	135.9	17.6	19.7	36.3	71.4	8.0	28.2	5.4	1.1	4.6	0.8	4.3	0.9	2.6	0.5	2.9	0.5	4.2	1.4	19.5
TGL15	21.2	4431.8	178.0	28.7	200.1	23.0	128.8	16.9	18.6	39.4	74.9	8.4	29.0	5.3	1.0	4.4	0.7	4.1	0.9	2.5	0.4	2.7	0.4	4.1	1.4	19.7
UCC	11.0	3000.0	60.0	17.0	112.0	22.0	190.0	25.0	3.7	30.0	64.0	7.1	26.0	4.5	0.9	3.8	0.6	3.5	0.8	2.3	0.3	2.2	0.5	5.8	2.2	10.5

Table S4

Table S4 REEs and hafnium concentrations(μg/g) of tree different grain size

Sample	**La**	**Ce**	**Pr**	Nd	**Sm**	**Eu**	**Gd**	**Tb**	**Dy**	**Ho**	**Er**	**Tm**	**Yb**	**Lu**	**Hf**
<0.45μm	33.91	67.53	6.10	21.72	4.40	1.07	4.49	0.83	4.93	1.07	3.12	0.54	3.31	0.54	3.91
0.45-2μm	39.42	74.95	8.37	28.96	5.34	1.03	4.45	0.75	4.11	0.85	2.52	0.43	2.72	0.45	4.08
>2μm	16.45	29.60	3.03	11.49	2.49	0.71	2.42	0.48	3.02	0.66	2.04	0.38	2.42	0.41	12.22
PAAS	38.20	79.60	8.83	33.09	5.55	1.08	4.66	0.77	4.68	0.99	2.85	0.41	2.82	0.43	1.43

Figure Captions:

Figure S1. Variation of ε_{Hf} values with the particle sizes.

Figure S2 The UCC standard cure for clay-sized fraction extracted from sands of Chinese deserts and Mongolian Gobi desert. Trace elements concentrations including REEs are normalized to UCC(Upper Crust, Taylor and McLennan, 1985) abundances, blue polygon is on behalf of the UCC-normalized patterns for the clay-sized fractions from Chinese deserts and Mongolian Gobi desert. Nonnormalized trace and REE elements concentrations are given in Table S3.

Figure S3 The PAAS standard curve for tree different grain size. The REEs concentrations and Hf concentration are normalized to PAAS (Post-Archaean Average Shale, (McLennan, 2001;). Nonnormalized hafnium and REE elements concentrations are given in Table S4.

Figure S1

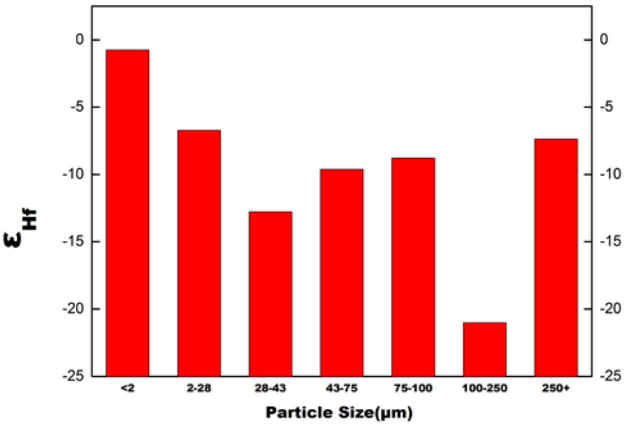

Figure S2

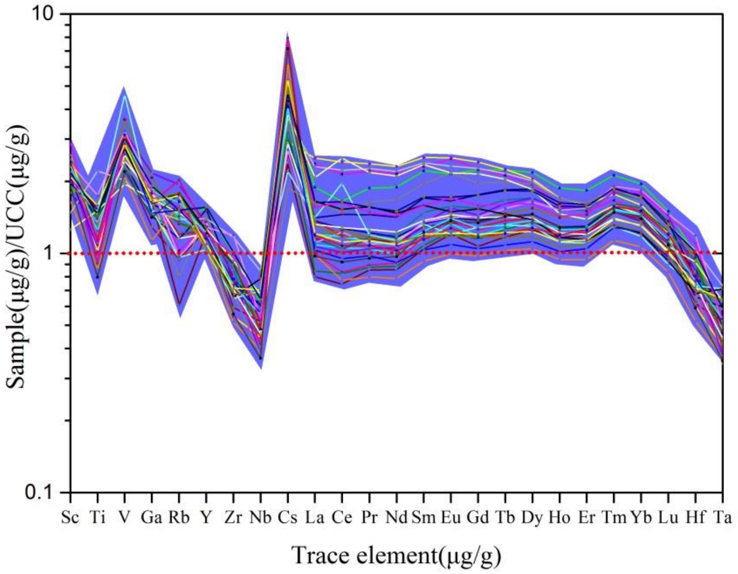

Figure S3

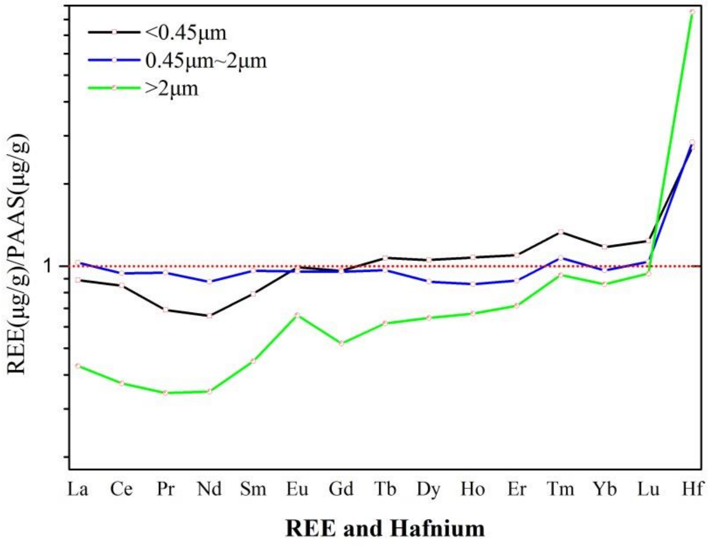

Supplement: Supplementary Information [file srep05837-s1.pdf]
